# Crystal structure and Hirshfeld surface analysis of 4-cyano-*N*-[(4-cyano­phen­yl)sulfon­yl]-*N*-[2-(5-methyl­furan-2-yl)phen­yl]benzene­sulfonamide

**DOI:** 10.1107/S2056989023006254

**Published:** 2023-07-21

**Authors:** Gunay Z. Mammadova, Elizaveta D. Yakovleva, Gleb M. Burkin, Victor N. Khrustalev, Mehmet Akkurt, Sevim Türktekin Çelikesir, Ajaya Bhattarai

**Affiliations:** a Organic Chemistry Department, Baku State University, Z. Xalilov Str. 23, Az 1148 Baku, Azerbaijan; b Peoples’ Friendship University of Russia (RUDN University), 6 Miklukho-Maklaya St., Moscow, 117198, Russian Federation; c Zelinsky Institute of Organic Chemistry of RAS, 4, 7 Leninsky Prospect, 119991 Moscow, Russian Federation; d Department of Physics, Faculty of Sciences, Erciyes University, 38039 Kayseri, Türkiye; e Department of Chemistry, M.M.A.M.C (Tribhuvan University), Biratnagar, Nepal; University of Neuchâtel, Switzerland

**Keywords:** crystal structure, sulfonamides, hydrogen bonds, C—H⋯π inter­actions, π–π inter­actions, Hirshfeld surface analysis

## Abstract

In the title compound, C_25_H_17_N_3_O_5_S_2_, intra­molecular π–π inter­actions [centroid-to-centroid distance = 3.5640 (9) Å] are observed between the furan and benzene rings of the 4-cyano­phenyl group. In the crystal, mol­ecules are connected *via* C—H⋯O and C—H⋯N hydrogen bonds, forming layers parallel to the (100) plane. These layers are inter­connected by C—H⋯π inter­actions and weak van der Waals inter­actions. Hirshfeld surface analysis indicates that H⋯H (30.2%), N⋯H/H⋯N (22.3%), C⋯H/H⋯C (17.9%) and O⋯H/H⋯O (15.4%) inter­actions make the most significant contributions to the crystal packing.

## Abstract 2

The crystal structure features C—H⋯O and C—H⋯N hydrogen bonds, which link the mol­ecules into layers parallel to the (100) plane. IC—H⋯π inter­actions and weak van der Waals inter­actions occur between the layers.

## 1. Chemical context

**Figure 1:**
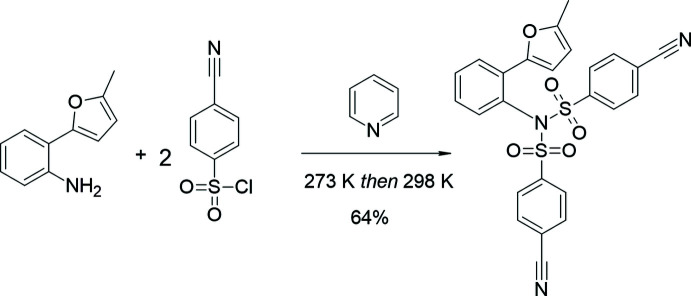
Reaction scheme showing the one-pot synthesis of the title compound.

The famous Hinsberg reaction, first described by Oscar Hinsberg in 1890 (Hinsberg, 1890 ▸; Hinsberg & Kessler, 1905 ▸), is a laboratory test used for the detection of primary, secondary and tertiary amines. In this reaction, the corres­ponding amine is shaken with benzyl or *p*-tolyl­sulfonyl chloride in the presence of an aqueous base. Reactions with ammonia, and primary and secondary amines are the most widespread. A primary amine will form a soluble sulfonamide salt in the presence of aqueous alkali (either KOH or NaOH). A secondary amine in the same reaction forms an insoluble sulfonamide. The most widely used sulfonyl­amide is sulfanil­amide, an anti­bacterial drug that was first obtained in 1908 by the Austrian chemist Paul Josef Jakob Gelmo while he was trying to synthesize a dye for textile materials (Gelmo, 1908 ▸). Moreover, sulfonyl­amides are active against seizures (Thiry *et al.*, 2008 ▸), and inhibit various enzymes such as human leukocyte elastase, cathespin G and HIV-1 protease (Supuran *et al.*, 2003 ▸). Sulfonamides are also used in fungicidal (Chohan *et al.*, 2006 ▸, 2010 ▸) and insecticidal mixtures (Beesley & Peters, 1971 ▸). The number of donor and acceptor groups is a fundamental mol­ecular descriptor to predict the oral bioavailability as well as biocatalytic activity of small drug candidates (Gurbanov *et al.*, 2020*a* ▸,*b* ▸, 2022 ▸; Mahmoudi *et al.*, 2017*a* ▸,*b* ▸). Continuing our research in the improved multiple displacement amplification (IMDA) reaction field (Mammadova *et al.*, 2023 ▸; Krishna *et al.*, 2022 ▸; Yarovaya *et al.*, 2021 ▸), in this work we have studied the inter­action of 2-(α-fur­yl)aniline with sulfochloride containing an electron-withdrawing 4-cyano­phenyl group. Unexpectedly, under mild reaction conditions, the product of a double sulfaryl­ation was isolated in good yield from the reaction mixture (Fig. 1 ▸). The formation of such double sulfonamide was previously observed in the presence of strong bases (Bartsch *et al.*, 1977 ▸; Li *et al.*, 2022 ▸). 
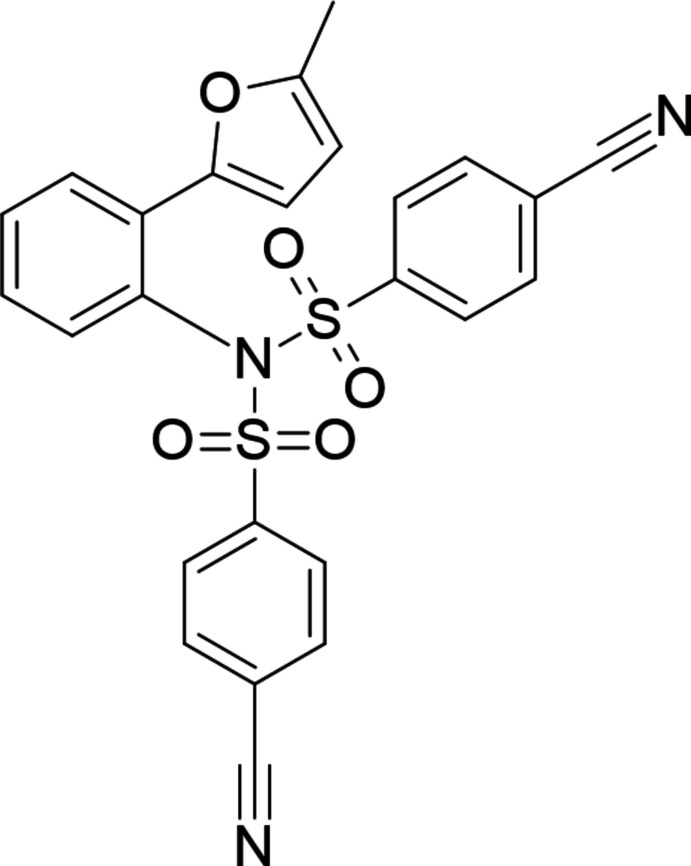


## 2. Structural commentary

**Figure 2:**
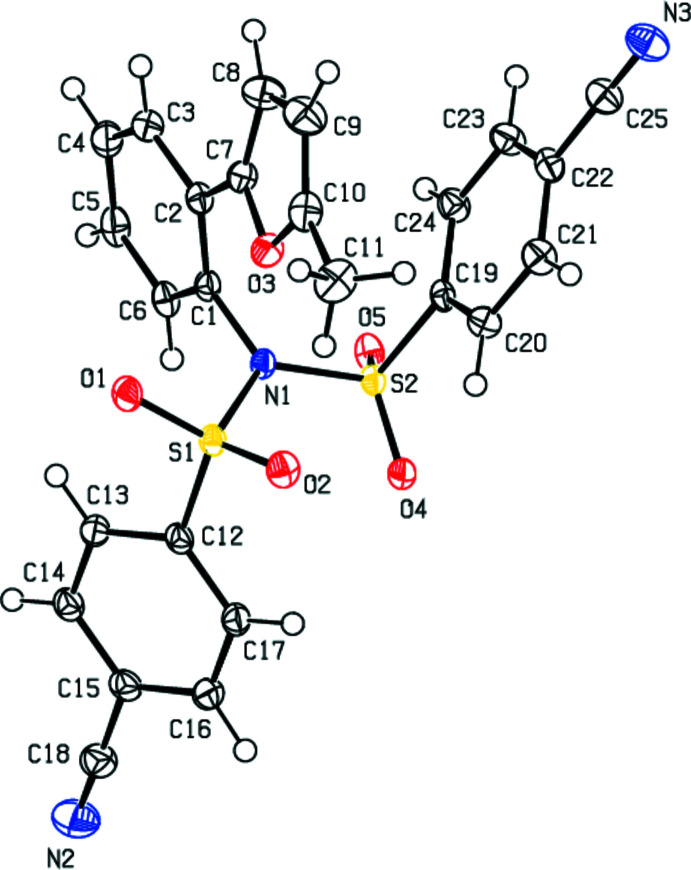
Mol­ecular structure of the title compound showing the atomic labelling. Displacement ellipsoids are drawn at the 50% probability level.

In the title compound (Fig. 2 ▸), the angle between the planes of the phenyl rings (C12–C17 and C19–C24) of the (4-cyano­phen­yl)sulfonyl groups is 47.90 (7)°. The furan ring (O1/C7–C10) is inclined at angles of 39.05 (8) and 17.38 (8)° with respect to the C12–C17 and C19–C24 phenyl rings of the (4-cyano­phen­yl)sulfonyl groups, while it makes a dihedral angle of 20.21 (8)° with the plane of the phenyl ring (C1–C6) attached to the furan ring. The latter phenyl ring makes dihedral angles of 26.28 (7) and 36.40 (7)°, respectively, with the phenyl rings of the (4-cyano­phen­yl)sulfonyl groups. All geometric parameters are normal and consistent with those of related compounds listed in the *Database survey* (Section 4).

Intra­molecular π–π stacking inter­actions [*Cg*1⋯*Cg*4 = 3.5640 (9) Å; *Cg*1 and *Cg*4 are the centroids of the furan (O1/C7–C10) and benzene rings (C19–C24), respectively, of one of the two 4-cyano­phen­yl)sulfonyl groups, respectively; slippage = 0.793 Å], ensures the stability of the mol­ecular configuration.

## 3. Supra­molecular features and Hirshfeld surface analysis

**Figure 3:**
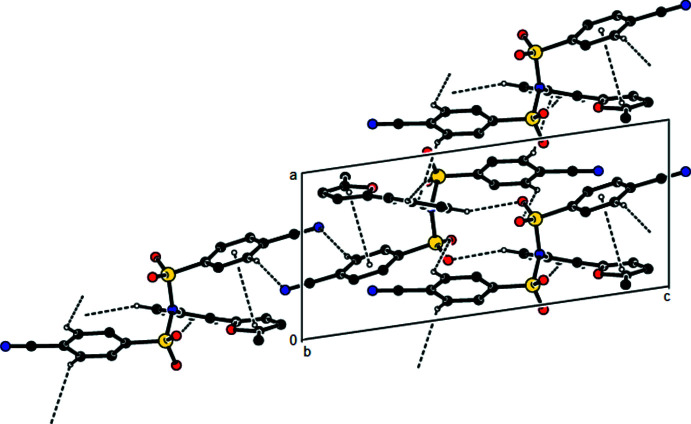
Crystal packing of the title compound along the *b* axis showing the C—H⋯O and C—H⋯N hydrogen bonds and C—H⋯π and π–π inter­actions.

In the crystal, mol­ecules are linked *via* C—H⋯O and C—H⋯N hydrogen bonds, forming layers parallel to the (100) plane (Table 1 ▸; Fig. 3 ▸). These layers are inter­connected by C—H⋯π inter­actions and weak van der Waals inter­actions, thus ensuring crystal cohesion.

**Figure 4:**
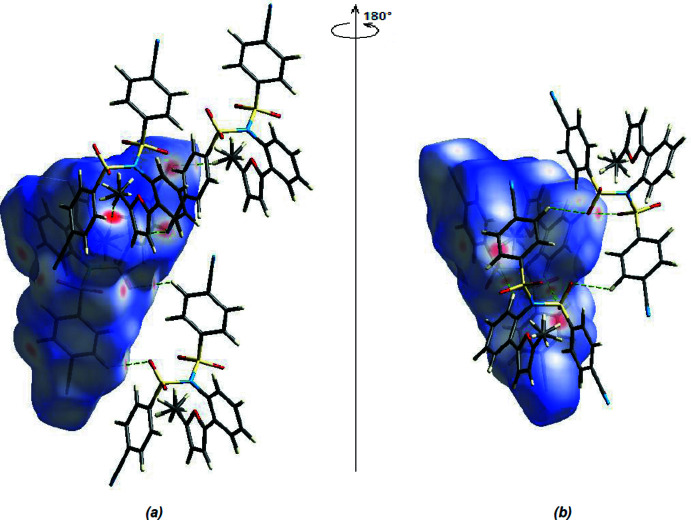
Front (*a*) and back (*b*) views of the three-dimensional Hirshfeld surface for the title compound. Some inter­molecular C—H⋯O and C—H⋯N inter­actions are shown.

**Figure 5:**
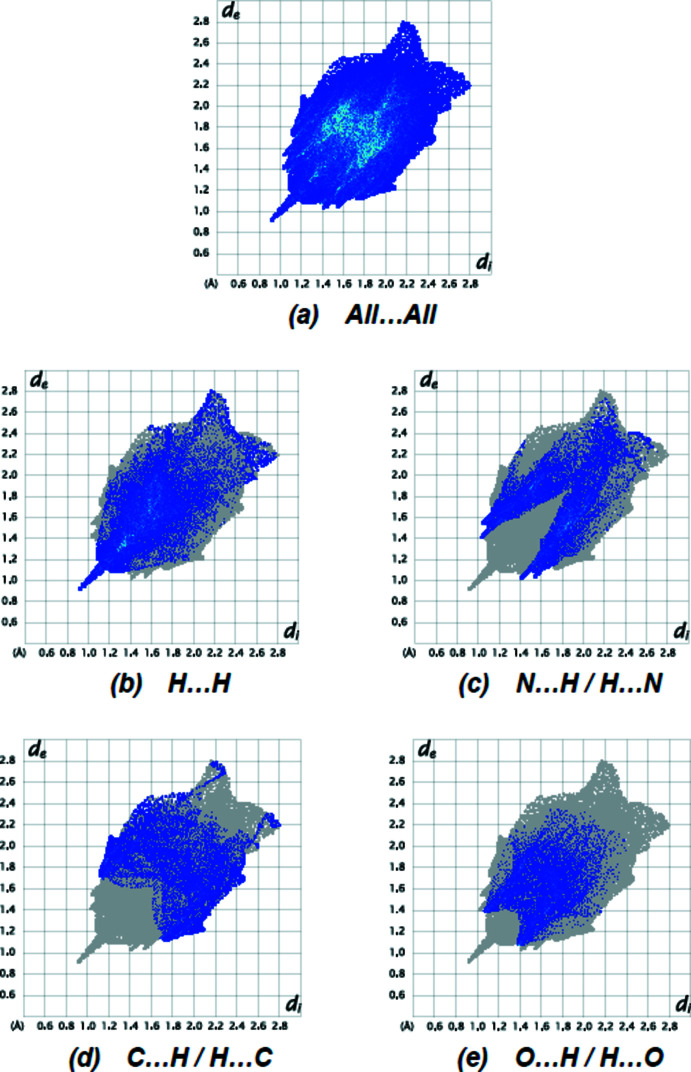
The two-dimensional fingerprint plots for the title compound showing (*a*) all inter­actions, and delineated into (*b*) H⋯H, (*c*) N⋯H/H⋯N, (*d*) C⋯H/H⋯C and (*e*) O⋯H/H⋯O inter­actions. The *d*_i_ and *d*_e_ values are the closest inter­nal and external distances (in Å) from given points on the Hirshfeld surface.

Hirshfeld surfaces were generated for the mol­ecule of the title compound using *Crystal Explorer 17.5* (Spackman *et al.*, 2021 ▸). The *d*_norm_ mappings was performed in the range −0.3260 to +1.4294 a.u. The C—H⋯O and C—H⋯N inter­actions are indicated by red areas on the Hirshfeld surfaces (Fig. 4 ▸). Fingerprint plots (Fig. 5 ▸) reveal that while H⋯H inter­actions (30.2%) make the largest contributions to surface contacts (Tables 1 ▸ and 2 ▸), N⋯H/H⋯N (22.3%), C⋯H/H⋯C (17.9%) and O⋯H/H⋯O (15.4%) contacts are also important. Other, less notable inter­actions are O⋯C/C⋯O (6.0%), C⋯C (5.0%), N⋯N (1.2%), O⋯O (1.1%), N⋯C/C⋯N (0.5%), S⋯H/H⋯S (0.1%) and S⋯O/O⋯S (0.1%).

## 4. Database survey

Ten related compounds were found as a result of the search for ‘*N-(methane­sulfon­yl)-N-methyl­methane­sulfonamide*’ in the Cambridge Structural Database (CSD, Version 5.42, update of September 2021; Groom *et al.*, 2016 ▸), *viz.* PIMGUR (Mammadova *et al.*, 2023 ▸), JOBTIF (Kim, 2014 ▸), CEGMIM (Mughal *et al.*, 2012*a* ▸), CEGSUE (Mughal *et al.*, 2012*b* ▸), YAXKAL (Taher & Smith, 2012*a* ▸), EFASUB (Taher & Smith, 2012*b* ▸), OCABUR (Abbassi *et al.*, 2011 ▸), AYUPUG (Arshad *et al.*, 2011 ▸), PONZIC (Rizzoli *et al.*, 2009 ▸) and ROGJON (Li & Song, 2008 ▸).

In PIMGUR (space group *P*2_1_/*n*), C—H⋯O hydrogen bonds link adjacent mol­ecules in a three-dimensional network, while π–π stacking inter­actions [centroid–centroid distance = 3.8745 (9) Å] between the furan and a phenyl ring of one of the two (3-nitro­phen­yl)sulfonyl groups result in chains parallel to the *a* axis. In JOBTIF (space group *P*2_1_/*n*), mol­ecules are linked by pairs of C—H⋯O hydrogen bonds, forming inversion dimers. In CEGMIM (space group *Pbca*), mol­ecules are connected by C—H⋯O inter­actions into sheets in the *ab* plane. In the crystal of CEGSUE (space group *P* 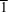 ), the only directional inter­actions are very weak C—H⋯π inter­actions and very weak π–π stacking between parallel methyl­phenyl rings. In YAXKAL (space group *P* 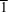 ), mol­ecules associate *via* pairs of N—H⋯N hydrogen bonds, forming a centrosymmetric eight-membered {⋯HNCN}_2_ synthon. In EFASUB (space group *C*2/*c*), mol­ecules associate *via* N—H⋯N and N—H⋯O hydrogen bonds, forming extended hydrogen-bonded sheets that lie parallel to the *bc* plane. The N—H⋯N hydrogen bonds propagate along the *b*-axis direction, while the N—H⋯O hydrogen bonds propagate along the *c*-axis direction. The crystal structure of OCABUR (space group *P*2_1_/*c*) features C—H⋯O hydrogen bonds. In the crystal structure of AYUPUG (space group *P*2_1_/*c*), weak C—H⋯O inter­actions connect the mol­ecules in a zigzag manner along the *a-*axis direction. In the crystal of PONZIC (space group *P* 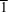 ), mol­ecules are linked into chains parallel to the *a* axis by inter­molecular C—H⋯O hydrogen bonds and π–π stacking inter­actions. In ROGJON (space group *Pbca*), the crystal structure features weak inter­molecular N—H⋯O, C—H⋯O and C—H⋯N hydrogen bonds and π–π inter­actions.

## 5. Synthesis and crystallization

*p-*Cyano­benzene­sulphonyl chloride (2.33 g, 0.0115 mol) was added gradually to a solution of 2-(5-methyl-2-fur­yl)aniline (1.00 g, 0.00577 mol) in pyridine (7 mL) under stirring and cooling in an ice–water bath. The mixture was stirred for 7 h at r.t. and after completion of the reaction [thin-layer chromatography (TLC) monitoring; sorbfil, hexa­ne/ethyl acetate 4:1], the mixture was poured into hydro­chloric acid (6 *M*, 90 mL). The separated oil was washed with water until its crystallization. The obtained crystals were filtered off, dried, and recrystallized from an ethanol/di­methyl­formamide (DMF) mixture to give the target disulfonamide as a colourless solid. Single crystals were obtained by slow crystallization from an EtOH/DMF mixture (yield 64%, 1.86 g; m.p. 507–508 K). IR (KBr), ν (cm^−1^): 1156 (ν_s_ SO_2_), 1329 (ν_as_ SO_2_), 2237 (CN). ^1^H NMR (600.2 MHz, DMSO-*d*_6_) (*J*, Hz): *δ* 8.08 (*d*, *J =* 8.6 Hz, 4H), 7.90 (*d*, *J =* 8.6 Hz, 4H), 7.72 (*dd*, *J =* 8.1, 1.5 Hz, 1H), 7.56 (*dt*, *J =* 8.6, 1.5 Hz, 1H), 7.36 (*dt*, *J =* 8.1, 1.5 Hz, 1H), 7.04 (*dd*, *J =* 8.1, 1.5 Hz, 1H), 6.61 (*d*, *J =* 3.5 Hz, 1H), 5.93 (*br.d*, *J* = 3.5 Hz, 1H), 1.96 (*s*, 3H); ^13^C{^1^H} NMR (150.9 MHz, DMSO-*d*_6_): *δ* 153.2, 147.9 (2C), 142.9, 134.0 (4C), 133.7, 132.2, 132.0, 129.6 (4C), 128.8, 128.7, 128.6, 117.9 (2C), 117.4 (2C), 112.0, 108.6, 13.4; MS (ESI) *m*/*z*: [*M* + H]^+^ 504. Elemental analysis calculated (%) for C_25_H_17_N_3_O_5_S_2_ %: C 59.63, H 3.40, N 8.34, S 12.74; found: C 60.00, H 3.27, N 8.56, S 13.03.

## 6. Refinement

Crystal data, data collection and structure refinement details are summarized in Table 3 ▸. All C-bound H atoms were positioned geometrically (C—H = 0.95 and 0.98 Å) and included as riding contributions with isotropic displacement parameters fixed at 1.2*U*_eq_(C) (1.5 for methyl groups). The hydrogen atoms of the methyl group containing the C11 atom were disordered over two positions with equal occupancies.

## Supplementary Material

Crystal structure: contains datablock(s) I. DOI: 10.1107/S2056989023006254/tx2071sup1.cif

Structure factors: contains datablock(s) I. DOI: 10.1107/S2056989023006254/tx2071Isup2.hkl

Supporting information file. DOI: 10.1107/S2056989023006254/tx2071Isup3.cml

CCDC reference: 2282070

Additional supporting information:  crystallographic information; 3D view; checkCIF report

## Figures and Tables

**Table 1:** Hydrogen-bond geometry (Å, °) *Cg*2 is the centroid of the ring.

*D*—H⋯*A*	*D*—H	H⋯*A*	*D*⋯*A*	*D*—H⋯*A*
C4—H4⋯O2^i^	0.95	2.56	3.3639 (17)	142
C6—H6⋯O5^ii^	0.95	2.56	3.3744 (17)	143
C11—H11*B*⋯N3^iii^	0.98	2.67	3.616 (2)	163
C16—H16⋯O4^iv^	0.95	2.55	3.2115 (18)	127
C21—H21⋯N3^iii^	0.95	2.54	3.433 (2)	156
C14—H14⋯*Cg*2^v^	0.95	2.85	3.4945 (16)	126

Symmetry codes: (i) 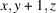 ; (ii) 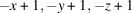 ; (iii) 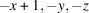 ; (iv) 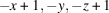 ; (v) 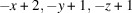 .

**Table 2:** Summary of short inter­atomic contacts (Å) in the title compound

Contact	Distance	Symmetry operation
C25⋯H11*C*	2.91	−1 + *x*, *y*, *z*
H20⋯H4	2.37	*x*, −1 + *y*, *z*
H11*E*⋯N2	2.76	2 − *x*, −*y*, 1 − *z*
H20⋯H16	2.44	1 − *x*, −*y*, 1 − *z*
H6⋯O5	2.56	1 − *x*, 1 − *y*, 1 − *z*
H21⋯N3	2.54	1 − *x*, −*y*, −*z*
H8⋯N3	2.73	1 − *x*, 1 − *y*, −*z*
H13⋯H13	2.40	2 − *x*, 1 − *y*, 1 − *z*
H11*D*⋯H11*D*	2.02	2 − *x*, −*y*, −*z*

**Table 3:** Experimental details

Crystal data	
Chemical formula	C_25_H_17_N_3_O_5_S_2_
*M*_r_	503.53
Crystal system, space group	Triclinic, *P* 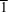
Temperature (K)	100
*a*, *b*, *c* (Å)	7.4542 (1), 9.5111 (2), 16.4378 (3)
α, β, γ (°)	88.838 (4), 81.644 (1), 81.414 (1)
*V* (Å^3^)	1140.11 (4)
*Z*	2
Radiation type	Cu *K*α
μ (mm^−1^)	2.50
Crystal size (mm)	0.29 × 0.22 × 0.15

Data collection	
Diffractometer	XtaLAB Synergy, Dualflex, HyPix
Absorption correction	Multi-scan (*CrysAlis PRO*; Rigaku OD, 2021 ▸)
*T*_min_, *T*_max_	0.481, 1.000
No. of measured, independent and observed [*I* > 2σ(*I*)] reflections	29936, 4841, 4675
*R*_int_	0.051
(sin θ/λ)_max_ (Å^−1^)	0.634

Refinement	
*R*[*F* ^2^ > 2σ(*F* ^2^)], *wR*(*F* ^2^), *S*	0.036, 0.095, 1.08
No. of reflections	4841
No. of parameters	316
H-atom treatment	H-atom parameters constrained
Δρ_max_, Δρ_min_ (e Å^−3^)	0.76, −0.60

Computer programs: *CrysAlis PRO* (Rigaku OD, 2021 ▸), *SHELXT2016/6* (Sheldrick, 2015*a* ▸), *SHELXL2016/6* (Sheldrick, 2015*b* ▸), *ORTEP-3 for Windows* (Farrugia, 2012 ▸) and *PLATON* (Spek, 2020 ▸).

## supplementary crystallographic information

### Crystal data

**Table:**

C_25_H_17_N_3_O_5_S_2_	*Z* = 2
*M**_r_* = 503.53	*F*(000) = 520
Triclinic, *P*1	*D*_x_ = 1.467 Mg m^−^^3^
*a* = 7.4542 (1) Å	Cu *K*α radiation, λ = 1.54184 Å
*b* = 9.5111 (2) Å	Cell parameters from 21520 reflections
*c* = 16.4378 (3) Å	θ = 2.7–77.6°
α = 88.838 (4)°	µ = 2.50 mm^−^^1^
β = 81.644 (1)°	*T* = 100 K
γ = 81.414 (1)°	Prism, colourless
*V* = 1140.11 (4) Å^3^	0.29 × 0.22 × 0.15 mm

### Data collection

**Table:**

XtaLAB Synergy, Dualflex, HyPix diffractometer	4841 independent reflections
Radiation source: micro-focus sealed X-ray tube	4675 reflections with *I* > 2σ(*I*)
Detector resolution: 0 pixels mm^-1^	*R*_int_ = 0.051
φ and ω scans	θ_max_ = 77.9°, θ_min_ = 2.7°
Absorption correction: multi-scan (CrysAlisPro; Rigaku OD, 2021)	*h* = −8→9
*T*_min_ = 0.481, *T*_max_ = 1.000	*k* = −12→12
29936 measured reflections	*l* = −20→20

### Refinement

**Table:**

Refinement on *F*^2^	0 restraints
Least-squares matrix: full	Hydrogen site location: inferred from neighbouring sites
*R*[*F*^2^ > 2σ(*F*^2^)] = 0.036	H-atom parameters constrained
*wR*(*F*^2^) = 0.095	*w* = 1/[σ^2^(*F*_o_^2^) + (0.0547*P*)^2^ + 0.4479*P*] where *P* = (*F*_o_^2^ + 2*F*_c_^2^)/3
*S* = 1.08	(Δ/σ)_max_ < 0.001
4841 reflections	Δρ_max_ = 0.76 e Å^−^^3^
316 parameters	Δρ_min_ = −0.60 e Å^−^^3^

### Special details

**Table:**

Experimental. CrysAlisPro 1.171.41.117a (Rigaku OD, 2021) Empirical absorption correction using spherical harmonics, implemented in SCALE3 ABSPACK scaling algorithm.
Geometry. All esds (except the esd in the dihedral angle between two l.s. planes) are estimated using the full covariance matrix. The cell esds are taken into account individually in the estimation of esds in distances, angles and torsion angles; correlations between esds in cell parameters are only used when they are defined by crystal symmetry. An approximate (isotropic) treatment of cell esds is used for estimating esds involving l.s. planes.

### Fractional atomic coordinates and isotropic or equivalent isotropic displacement parameters (Å^2^)

**Table:**

	*x*	*y*	*z*	*U*_iso_*/*U*_eq_	Occ. (<1)
S1	0.87131 (4)	0.20718 (3)	0.37143 (2)	0.01631 (10)	
S2	0.47488 (4)	0.28580 (3)	0.36436 (2)	0.01531 (10)	
O1	0.86050 (14)	0.27336 (10)	0.19206 (6)	0.0209 (2)	
O2	0.85236 (14)	0.07100 (10)	0.34200 (6)	0.0224 (2)	
O3	1.02688 (13)	0.27486 (11)	0.34195 (6)	0.0214 (2)	
O4	0.47897 (13)	0.15314 (11)	0.40679 (6)	0.0207 (2)	
O5	0.35697 (13)	0.40880 (11)	0.39862 (6)	0.0208 (2)	
N1	0.68945 (15)	0.32549 (12)	0.35205 (7)	0.0154 (2)	
N2	0.7640 (2)	0.22944 (17)	0.80753 (9)	0.0376 (3)	
N3	0.3238 (2)	0.18901 (15)	−0.04558 (8)	0.0301 (3)	
C1	0.71285 (17)	0.47104 (14)	0.33138 (8)	0.0166 (3)	
C2	0.76977 (18)	0.51151 (15)	0.25007 (9)	0.0190 (3)	
C3	0.7802 (2)	0.65682 (16)	0.23618 (10)	0.0244 (3)	
H3	0.819030	0.687961	0.182071	0.029*	
C4	0.7355 (2)	0.75524 (15)	0.29928 (10)	0.0261 (3)	
H4	0.742788	0.852707	0.287872	0.031*	
C5	0.67996 (19)	0.71301 (16)	0.37940 (10)	0.0238 (3)	
H5	0.649904	0.780898	0.422687	0.029*	
C6	0.66904 (18)	0.57071 (15)	0.39520 (9)	0.0196 (3)	
H6	0.631585	0.540682	0.449686	0.024*	
C7	0.81504 (19)	0.41619 (15)	0.17921 (9)	0.0201 (3)	
C8	0.8215 (2)	0.44092 (18)	0.09722 (10)	0.0294 (3)	
H8	0.796625	0.530602	0.071431	0.035*	
C9	0.8728 (2)	0.30600 (19)	0.05734 (9)	0.0308 (3)	
H9	0.888002	0.288669	−0.000144	0.037*	
C10	0.8957 (2)	0.20782 (17)	0.11670 (9)	0.0242 (3)	
C11	0.9513 (2)	0.05141 (18)	0.11806 (10)	0.0320 (4)	
H11A	0.951112	0.019245	0.175088	0.048*	0.5
H11B	0.864718	0.004742	0.092582	0.048*	0.5
H11C	1.074697	0.026944	0.087412	0.048*	0.5
H11D	0.975906	0.014709	0.061633	0.048*	0.5
H11E	1.062300	0.029212	0.144140	0.048*	0.5
H11F	0.852321	0.007010	0.149309	0.048*	0.5
C12	0.85015 (18)	0.20354 (14)	0.47982 (8)	0.0176 (3)	
C13	0.91148 (19)	0.31300 (14)	0.51825 (9)	0.0197 (3)	
H13	0.967389	0.383552	0.486494	0.024*	
C14	0.88981 (19)	0.31752 (15)	0.60333 (9)	0.0208 (3)	
H14	0.928202	0.392513	0.630580	0.025*	
C15	0.81124 (19)	0.21113 (15)	0.64858 (9)	0.0206 (3)	
C16	0.75614 (19)	0.09909 (15)	0.60938 (9)	0.0225 (3)	
H16	0.706667	0.025466	0.640954	0.027*	
C17	0.77392 (19)	0.09578 (15)	0.52430 (9)	0.0204 (3)	
H17	0.734758	0.021287	0.496928	0.025*	
C18	0.7855 (2)	0.21874 (17)	0.73718 (10)	0.0265 (3)	
C19	0.43219 (17)	0.26331 (14)	0.26310 (8)	0.0161 (3)	
C20	0.47823 (19)	0.12911 (15)	0.22776 (9)	0.0201 (3)	
H20	0.531585	0.051367	0.257755	0.024*	
C21	0.4455 (2)	0.10992 (15)	0.14829 (9)	0.0223 (3)	
H21	0.473180	0.018398	0.123514	0.027*	
C22	0.37117 (19)	0.22685 (16)	0.10504 (8)	0.0203 (3)	
C23	0.3246 (2)	0.36132 (16)	0.14114 (9)	0.0234 (3)	
H23	0.272363	0.439559	0.111171	0.028*	
C24	0.3553 (2)	0.37968 (15)	0.22120 (9)	0.0212 (3)	
H24	0.324252	0.470377	0.246872	0.025*	
C25	0.3437 (2)	0.20666 (16)	0.02113 (9)	0.0242 (3)	

### Atomic displacement parameters (Å^2^)

**Table:**

	*U*^11^	*U*^22^	*U*^33^	*U*^12^	*U*^13^	*U*^23^
S1	0.01287 (16)	0.01633 (16)	0.01966 (17)	−0.00131 (11)	−0.00276 (12)	−0.00148 (12)
S2	0.01201 (16)	0.01925 (17)	0.01516 (16)	−0.00367 (11)	−0.00191 (11)	−0.00204 (12)
O1	0.0234 (5)	0.0214 (5)	0.0178 (5)	−0.0057 (4)	0.0000 (4)	−0.0028 (4)
O2	0.0235 (5)	0.0175 (5)	0.0260 (5)	−0.0012 (4)	−0.0044 (4)	−0.0049 (4)
O3	0.0134 (5)	0.0265 (5)	0.0241 (5)	−0.0030 (4)	−0.0020 (4)	0.0003 (4)
O4	0.0192 (5)	0.0256 (5)	0.0193 (5)	−0.0086 (4)	−0.0045 (4)	0.0028 (4)
O5	0.0143 (4)	0.0260 (5)	0.0215 (5)	−0.0012 (4)	−0.0015 (4)	−0.0081 (4)
N1	0.0119 (5)	0.0163 (5)	0.0184 (5)	−0.0034 (4)	−0.0024 (4)	−0.0002 (4)
N2	0.0433 (9)	0.0437 (8)	0.0247 (7)	−0.0043 (7)	−0.0037 (6)	0.0032 (6)
N3	0.0361 (7)	0.0327 (7)	0.0207 (6)	0.0003 (6)	−0.0065 (5)	−0.0033 (5)
C1	0.0133 (6)	0.0157 (6)	0.0214 (6)	−0.0023 (4)	−0.0044 (5)	−0.0007 (5)
C2	0.0155 (6)	0.0206 (6)	0.0220 (7)	−0.0040 (5)	−0.0055 (5)	0.0013 (5)
C3	0.0247 (7)	0.0229 (7)	0.0279 (7)	−0.0066 (5)	−0.0095 (6)	0.0056 (6)
C4	0.0239 (7)	0.0174 (6)	0.0393 (9)	−0.0028 (5)	−0.0129 (6)	0.0026 (6)
C5	0.0187 (7)	0.0204 (7)	0.0333 (8)	−0.0010 (5)	−0.0080 (6)	−0.0072 (6)
C6	0.0148 (6)	0.0214 (7)	0.0231 (7)	−0.0017 (5)	−0.0049 (5)	−0.0037 (5)
C7	0.0170 (6)	0.0225 (7)	0.0211 (7)	−0.0046 (5)	−0.0021 (5)	0.0018 (5)
C8	0.0334 (8)	0.0322 (8)	0.0208 (7)	−0.0011 (6)	−0.0024 (6)	0.0030 (6)
C9	0.0332 (8)	0.0394 (9)	0.0178 (7)	−0.0019 (7)	−0.0002 (6)	−0.0035 (6)
C10	0.0222 (7)	0.0302 (8)	0.0195 (7)	−0.0065 (6)	0.0029 (5)	−0.0076 (6)
C11	0.0368 (9)	0.0291 (8)	0.0276 (8)	−0.0056 (6)	0.0054 (7)	−0.0087 (6)
C12	0.0139 (6)	0.0173 (6)	0.0218 (6)	−0.0001 (5)	−0.0056 (5)	0.0002 (5)
C13	0.0186 (6)	0.0180 (6)	0.0236 (7)	−0.0041 (5)	−0.0055 (5)	0.0016 (5)
C14	0.0190 (7)	0.0196 (6)	0.0244 (7)	−0.0010 (5)	−0.0071 (5)	−0.0005 (5)
C15	0.0159 (6)	0.0225 (7)	0.0227 (7)	0.0009 (5)	−0.0047 (5)	0.0025 (5)
C16	0.0184 (7)	0.0223 (7)	0.0276 (7)	−0.0042 (5)	−0.0055 (5)	0.0056 (5)
C17	0.0177 (6)	0.0180 (6)	0.0269 (7)	−0.0034 (5)	−0.0072 (5)	0.0012 (5)
C18	0.0242 (7)	0.0271 (7)	0.0279 (8)	−0.0023 (6)	−0.0050 (6)	0.0039 (6)
C19	0.0116 (6)	0.0210 (6)	0.0164 (6)	−0.0038 (5)	−0.0022 (5)	−0.0020 (5)
C20	0.0202 (7)	0.0196 (6)	0.0195 (6)	0.0001 (5)	−0.0027 (5)	−0.0007 (5)
C21	0.0243 (7)	0.0214 (7)	0.0205 (7)	−0.0006 (5)	−0.0028 (5)	−0.0055 (5)
C22	0.0167 (6)	0.0277 (7)	0.0169 (6)	−0.0042 (5)	−0.0026 (5)	−0.0022 (5)
C23	0.0257 (7)	0.0223 (7)	0.0224 (7)	−0.0004 (5)	−0.0078 (5)	0.0012 (5)
C24	0.0224 (7)	0.0197 (6)	0.0217 (7)	−0.0014 (5)	−0.0058 (5)	−0.0029 (5)
C25	0.0239 (7)	0.0272 (7)	0.0206 (7)	−0.0008 (6)	−0.0027 (5)	−0.0022 (6)

### Geometric parameters (Å, º)

**Table:**

S1—O2	1.4253 (10)	C10—C11	1.484 (2)
S1—O3	1.4287 (10)	C11—H11A	0.9800
S1—N1	1.6914 (11)	C11—H11B	0.9800
S1—C12	1.7657 (14)	C11—H11C	0.9800
S2—O5	1.4262 (10)	C11—H11D	0.9800
S2—O4	1.4277 (10)	C11—H11E	0.9800
S2—N1	1.6807 (11)	C11—H11F	0.9800
S2—C19	1.7625 (13)	C12—C17	1.3895 (19)
O1—C7	1.3699 (17)	C12—C13	1.3946 (19)
O1—C10	1.3711 (17)	C13—C14	1.385 (2)
N1—C1	1.4484 (16)	C13—H13	0.9500
N2—C18	1.148 (2)	C14—C15	1.393 (2)
N3—C25	1.147 (2)	C14—H14	0.9500
C1—C6	1.3979 (19)	C15—C16	1.398 (2)
C1—C2	1.4062 (19)	C15—C18	1.443 (2)
C2—C3	1.408 (2)	C16—C17	1.386 (2)
C2—C7	1.459 (2)	C16—H16	0.9500
C3—C4	1.382 (2)	C17—H17	0.9500
C3—H3	0.9500	C19—C24	1.3863 (19)
C4—C5	1.392 (2)	C19—C20	1.3877 (19)
C4—H4	0.9500	C20—C21	1.384 (2)
C5—C6	1.385 (2)	C20—H20	0.9500
C5—H5	0.9500	C21—C22	1.396 (2)
C6—H6	0.9500	C21—H21	0.9500
C7—C8	1.359 (2)	C22—C23	1.395 (2)
C8—C9	1.427 (2)	C22—C25	1.4442 (19)
C8—H8	0.9500	C23—C24	1.387 (2)
C9—C10	1.348 (2)	C23—H23	0.9500
C9—H9	0.9500	C24—H24	0.9500
			
O2—S1—O3	121.57 (6)	C10—C11—H11B	109.5
O2—S1—N1	108.74 (6)	H11A—C11—H11B	109.5
O3—S1—N1	104.43 (6)	C10—C11—H11C	109.5
O2—S1—C12	109.39 (6)	H11A—C11—H11C	109.5
O3—S1—C12	107.41 (6)	H11B—C11—H11C	109.5
N1—S1—C12	103.85 (6)	H11D—C11—H11E	109.5
O5—S2—O4	120.18 (6)	H11D—C11—H11F	109.5
O5—S2—N1	106.78 (6)	H11E—C11—H11F	109.5
O4—S2—N1	106.87 (6)	C17—C12—C13	121.92 (13)
O5—S2—C19	108.34 (6)	C17—C12—S1	120.72 (11)
O4—S2—C19	109.55 (6)	C13—C12—S1	117.36 (11)
N1—S2—C19	103.90 (6)	C14—C13—C12	119.13 (13)
C7—O1—C10	107.60 (11)	C14—C13—H13	120.4
C1—N1—S2	117.35 (9)	C12—C13—H13	120.4
C1—N1—S1	119.89 (9)	C13—C14—C15	119.42 (13)
S2—N1—S1	122.51 (7)	C13—C14—H14	120.3
C6—C1—C2	121.38 (13)	C15—C14—H14	120.3
C6—C1—N1	117.08 (12)	C14—C15—C16	120.97 (14)
C2—C1—N1	121.49 (12)	C14—C15—C18	118.91 (14)
C1—C2—C3	116.84 (13)	C16—C15—C18	120.11 (13)
C1—C2—C7	125.48 (12)	C17—C16—C15	119.79 (13)
C3—C2—C7	117.67 (13)	C17—C16—H16	120.1
C4—C3—C2	121.64 (14)	C15—C16—H16	120.1
C4—C3—H3	119.2	C16—C17—C12	118.71 (13)
C2—C3—H3	119.2	C16—C17—H17	120.6
C3—C4—C5	120.66 (14)	C12—C17—H17	120.6
C3—C4—H4	119.7	N2—C18—C15	177.81 (17)
C5—C4—H4	119.7	C24—C19—C20	122.18 (13)
C6—C5—C4	119.13 (14)	C24—C19—S2	119.24 (10)
C6—C5—H5	120.4	C20—C19—S2	118.57 (10)
C4—C5—H5	120.4	C21—C20—C19	119.19 (13)
C5—C6—C1	120.35 (14)	C21—C20—H20	120.4
C5—C6—H6	119.8	C19—C20—H20	120.4
C1—C6—H6	119.8	C20—C21—C22	119.10 (13)
C8—C7—O1	109.22 (13)	C20—C21—H21	120.5
C8—C7—C2	131.86 (14)	C22—C21—H21	120.5
O1—C7—C2	118.92 (12)	C23—C22—C21	121.32 (13)
C7—C8—C9	106.68 (14)	C23—C22—C25	120.03 (13)
C7—C8—H8	126.7	C21—C22—C25	118.65 (13)
C9—C8—H8	126.7	C24—C23—C22	119.37 (13)
C10—C9—C8	107.01 (13)	C24—C23—H23	120.3
C10—C9—H9	126.5	C22—C23—H23	120.3
C8—C9—H9	126.5	C19—C24—C23	118.81 (13)
C9—C10—O1	109.49 (13)	C19—C24—H24	120.6
C9—C10—C11	135.03 (14)	C23—C24—H24	120.6
O1—C10—C11	115.47 (13)	N3—C25—C22	179.03 (16)
C10—C11—H11A	109.5		
			
O5—S2—N1—C1	−33.62 (11)	C8—C9—C10—O1	0.37 (18)
O4—S2—N1—C1	−163.42 (10)	C8—C9—C10—C11	−178.47 (17)
C19—S2—N1—C1	80.78 (11)	C7—O1—C10—C9	−0.20 (16)
O5—S2—N1—S1	140.59 (8)	C7—O1—C10—C11	178.89 (13)
O4—S2—N1—S1	10.79 (9)	O2—S1—C12—C17	−16.82 (13)
C19—S2—N1—S1	−105.01 (8)	O3—S1—C12—C17	−150.62 (11)
O2—S1—N1—C1	−145.42 (10)	N1—S1—C12—C17	99.12 (12)
O3—S1—N1—C1	−14.26 (11)	O2—S1—C12—C13	163.63 (10)
C12—S1—N1—C1	98.17 (11)	O3—S1—C12—C13	29.83 (12)
O2—S1—N1—S2	40.51 (9)	N1—S1—C12—C13	−80.43 (11)
O3—S1—N1—S2	171.67 (7)	C17—C12—C13—C14	−2.4 (2)
C12—S1—N1—S2	−75.89 (9)	S1—C12—C13—C14	177.14 (10)
S2—N1—C1—C6	77.49 (14)	C12—C13—C14—C15	1.5 (2)
S1—N1—C1—C6	−96.87 (13)	C13—C14—C15—C16	0.8 (2)
S2—N1—C1—C2	−99.91 (13)	C13—C14—C15—C18	−178.59 (13)
S1—N1—C1—C2	85.72 (14)	C14—C15—C16—C17	−2.2 (2)
C6—C1—C2—C3	−0.1 (2)	C18—C15—C16—C17	177.20 (13)
N1—C1—C2—C3	177.24 (12)	C15—C16—C17—C12	1.3 (2)
C6—C1—C2—C7	−178.78 (13)	C13—C12—C17—C16	1.0 (2)
N1—C1—C2—C7	−1.5 (2)	S1—C12—C17—C16	−178.51 (10)
C1—C2—C3—C4	−0.4 (2)	O5—S2—C19—C24	23.09 (13)
C7—C2—C3—C4	178.39 (13)	O4—S2—C19—C24	155.90 (11)
C2—C3—C4—C5	0.6 (2)	N1—S2—C19—C24	−90.20 (12)
C3—C4—C5—C6	−0.3 (2)	O5—S2—C19—C20	−157.37 (11)
C4—C5—C6—C1	−0.2 (2)	O4—S2—C19—C20	−24.55 (13)
C2—C1—C6—C5	0.4 (2)	N1—S2—C19—C20	89.35 (11)
N1—C1—C6—C5	−177.05 (12)	C24—C19—C20—C21	−0.5 (2)
C10—O1—C7—C8	−0.07 (16)	S2—C19—C20—C21	180.00 (11)
C10—O1—C7—C2	179.71 (12)	C19—C20—C21—C22	1.6 (2)
C1—C2—C7—C8	158.84 (16)	C20—C21—C22—C23	−1.9 (2)
C3—C2—C7—C8	−19.9 (2)	C20—C21—C22—C25	177.80 (13)
C1—C2—C7—O1	−20.9 (2)	C21—C22—C23—C24	1.0 (2)
C3—C2—C7—O1	160.40 (12)	C25—C22—C23—C24	−178.67 (13)
O1—C7—C8—C9	0.29 (17)	C20—C19—C24—C23	−0.4 (2)
C2—C7—C8—C9	−179.45 (15)	S2—C19—C24—C23	179.11 (11)
C7—C8—C9—C10	−0.40 (19)	C22—C23—C24—C19	0.2 (2)

### Hydrogen-bond geometry (Å, º)

*Cg*2 is the centroid of the ring.

**Table:**

*D*—H···*A*	*D*—H	H···*A*	*D*···*A*	*D*—H···*A*
C4—H4···O2^i^	0.95	2.56	3.3639 (17)	142
C6—H6···O5^ii^	0.95	2.56	3.3744 (17)	143
C11—H11*B*···N3^iii^	0.98	2.67	3.616 (2)	163
C13—H13···O3	0.95	2.56	2.9146 (18)	102
C16—H16···O4^iv^	0.95	2.55	3.2115 (18)	127
C21—H21···N3^iii^	0.95	2.54	3.433 (2)	156
C24—H24···O5	0.95	2.59	2.9371 (18)	102
C14—H14···*Cg*2^v^	0.95	2.85	3.4945 (16)	126

Symmetry codes: (i) *x*, *y*+1, *z*; (ii) −*x*+1, −*y*+1, −*z*+1; (iii) −*x*+1, −*y*, −*z*; (iv) −*x*+1, −*y*, −*z*+1; (v) −*x*+2, −*y*+1, −*z*+1.
